# Valorization of a plant β-amylase: Immobilization and dataset on the kinetic process

**DOI:** 10.1016/j.dib.2017.11.071

**Published:** 2017-11-23

**Authors:** Imen Lahmar, Greta Radeva, Dessislava Marinkova, Maya Velitchkova, Hafedh Belghith, Ferjani Ben Abdallah, Lyubov Yotova, Karima Belghith

**Affiliations:** a Department of Biotechnology, University of Chemical Technology and Metallurgy, Sofia, Bulgaria; b Department of Physical Chemistry, University of Chemical Technology and Metallurgy, Sofia, Bulgaria; c Institute of Biophysics and Biomedical Engineering, Bulgarian Academy of Sciences, Sofia, Bulgaria; d Laboratory of Plant Biodiversity and Dynamics of Ecosystems in Arid Environment, Faculty of Sciences, Sfax, Tunisia; e Laboratory of Molecular Biotechnology of Eukaryotes, Center of Biotechnology, Sfax, Tunisia; f Laboratory of Plant Biotechnology Applied to Crop Improvement, Faculty of Sciences, Sfax, Tunisia

## Abstract

The data presented in this article are related to the research article titled “Immobilization nd topochemical mechanism of a new β-amylase extracted from Pergularia tomentosa” (Lahmar et al., 2017) [1]. This article documented information on the determination of the molecular weight of the β-amylase, the method of its immobilization and a comparison of the kinetic mechanism between the free and the immobilized forms by a mathematical method. Fresh Pergularia tomentosa was collected from Tunisia and a special method for β-amylase extraction was followed (Yotova et al., 2000) [2]. Public dissemination of this dataset will allow further analyses of the data.

**Specifications Table**

**Table:** 

Subject area	Biotechnology
More specific subject area	Plant Biology, Enzymology
Type of data	Tables, Figures, Text file
How data was acquired	absorption spectrophotometer and spectrophotometric methods as well mathematical calculations
Data format	Analyzed
Experimental factors	The whole plant treated with acetone and ethanol
Experimental features	Dried Pergularia tomentosa was defatted by maceration with acetone. Successive steps of extraction with ethanol were followed and the final pellet containing the amylase was dissolved in water.
Data source location	Melloulech /Tunisia, 35°09'08.7"N 11°01'35.1"E.
Data accessibility	The data are available with this article [1]

**Value of the data**

• Data can be implied in industrial sector in order to minimize the quantity of requested matrices retaining amylase activity.
• Data can be exploited in starch textile industries and liquefication of amylases.
• Data can permit other researchers to continue and extend the statistical analyses by comparing the kinetic mechanisms and clarifying the reaction processes between different plants.

## 1 Data

Data identify the molecular weight of the extracted β-amylase (see Fig. 1 in [1]). Data include the kinetic process of the conversion degree both of the free and the immobilized β-amylases at different temperatures (see Fig. 2 and 6 in [1]), in addition to linear form of Prout-tompkins equation (see Fig. 3 and 7 in [1]), the dependence of rate as a function of the conversion degree (see Fig. 4 and 8 in [1]) and the temperature dependence of the current rate and the rate constant at a constant degree of conversion (see Fig. 5 and 9 in [1]).

**Fig. 1:**
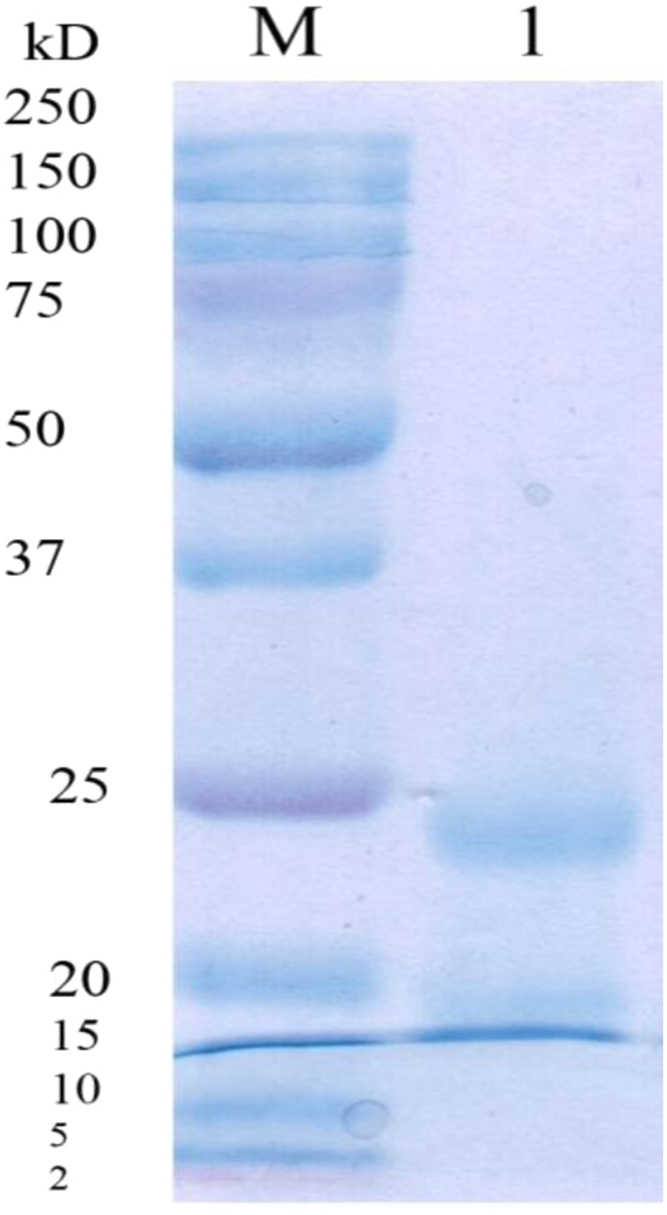
SDS-PAGE of proteins extracted from *Pergularia tomentosa* (1) in comparison with marker M.

**Fig. 2:**
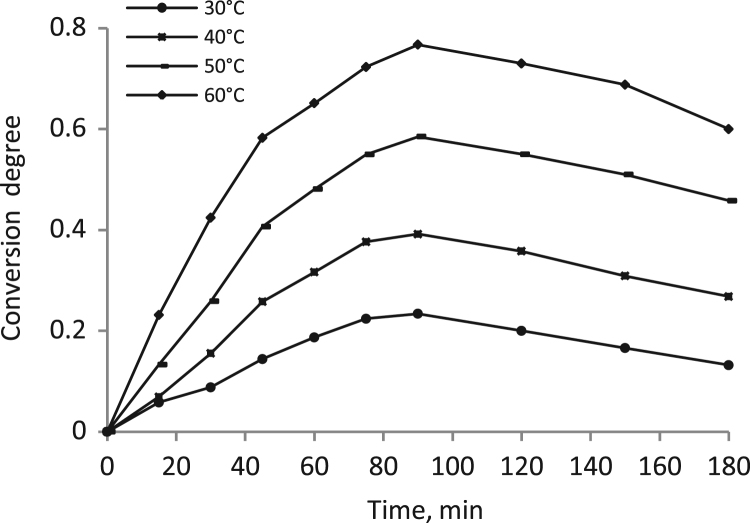
Kinetic curves of the process at the studied temperatures for the free β-amylase.

**Fig. 3:**
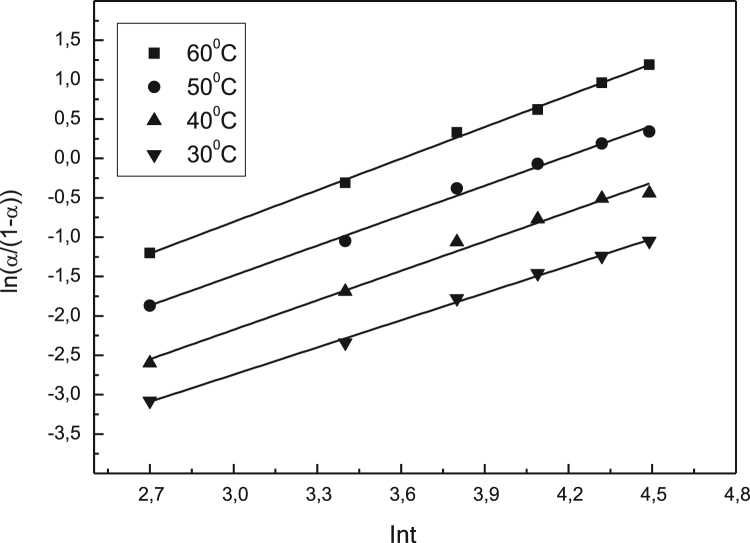
Linear form of Prout – Tompkins equation for different temperatures for the free β-amylase.

**Fig. 4:**
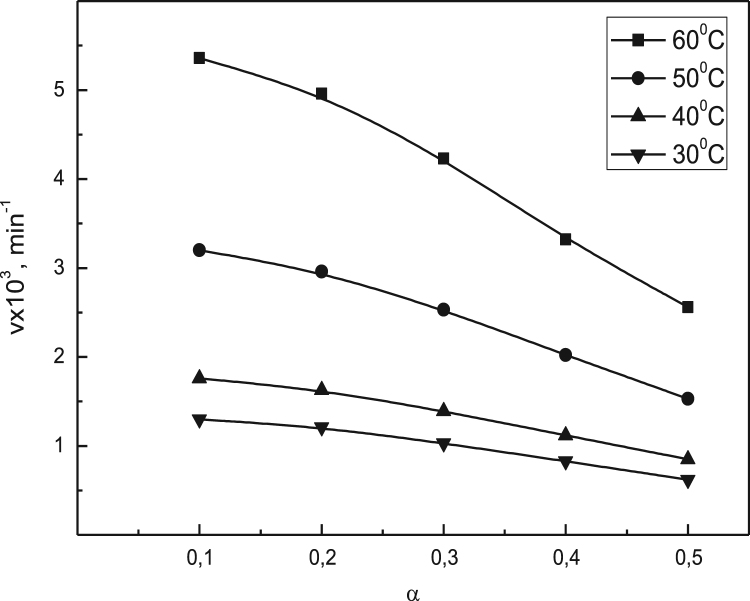
Dependence of rate *v*, mim^−1^ as a function of α at different temperatures for the free β-amylase.

**Fig. 5:**
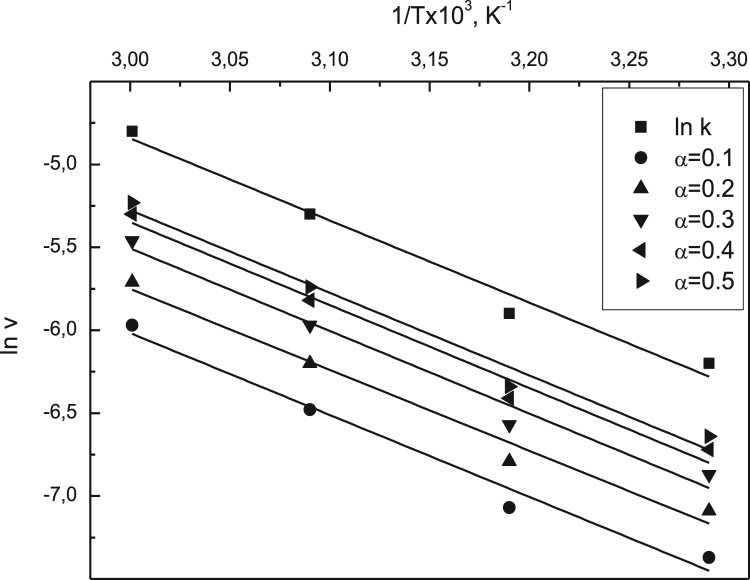
Temperature dependence of the current rate and the rate constant at α=*const* for the free β-amylase.

**Fig. 6:**
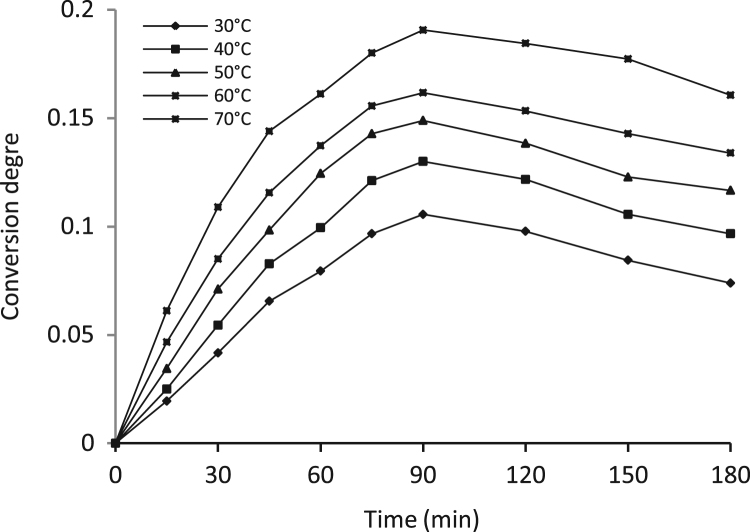
Kinetic curves of the process at the studied temperatures for the immobilized β-amylase.

**Fig. 7:**
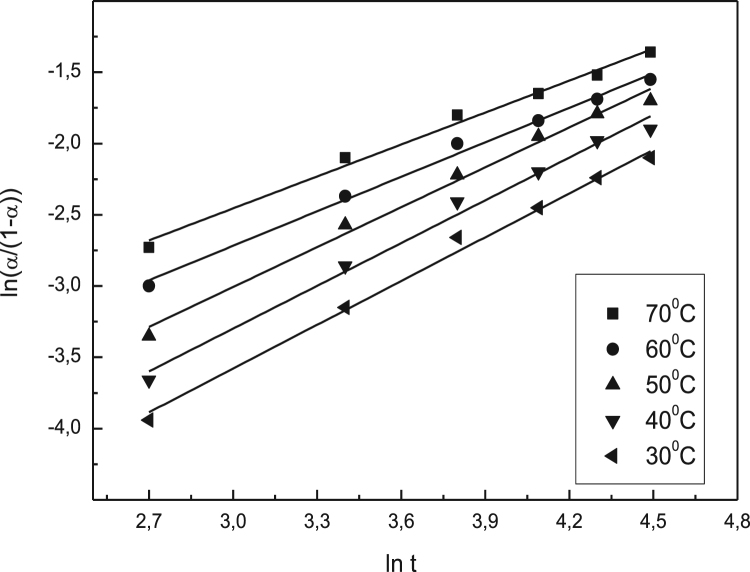
Linear form of Prout – Tompkins equation for different temperatures for the immobilized β-amylase.

## 2 Experimental design, materials and methods

*Pergularia tomentosa* was rinsed with water and kept to dry in darkness at room temperature. The dried and fine grounded plant was macerated with acetone. The defatted obtained Pergularia was macerated with 30% ethanol and then a series of centrifugation at 3000 rpm/min and precipitation of β-amylase was followed. The final obtained pellet was dissolved in water and it was considered as the plant extract [2]. The molecular weight of the extracted enzyme was determined in the presence of a marker in the range of 2–250 kD [3]. β-amylase was immobilized onto a butanol and titanium dioxide based matrix which is synthesized by incorporation of a polymer of cellulose acetate butyrate and copolymer of acrylonitrile and acrylamide to inorganic network [4], [5], [6] ().

**Fig. 8:**
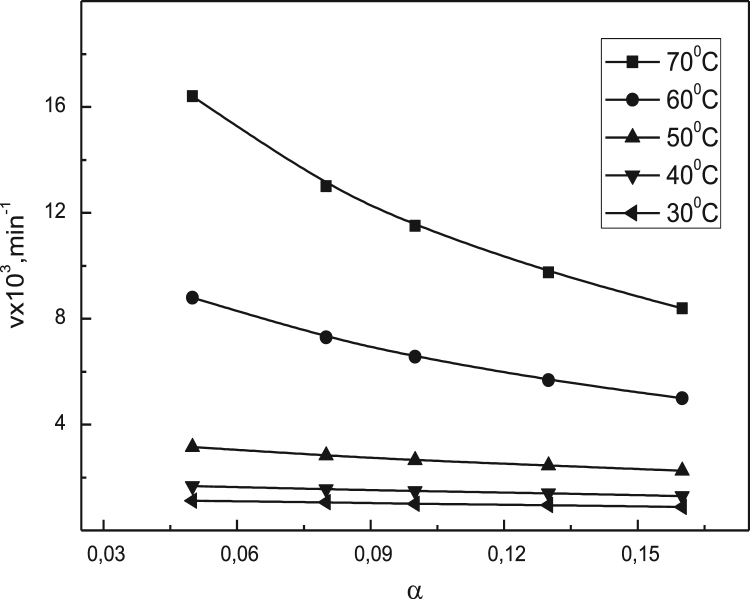
Dependence of rate *v*, mim^−1^ as a function of α at different temperatures for β-amylase.

**Fig. 9:**
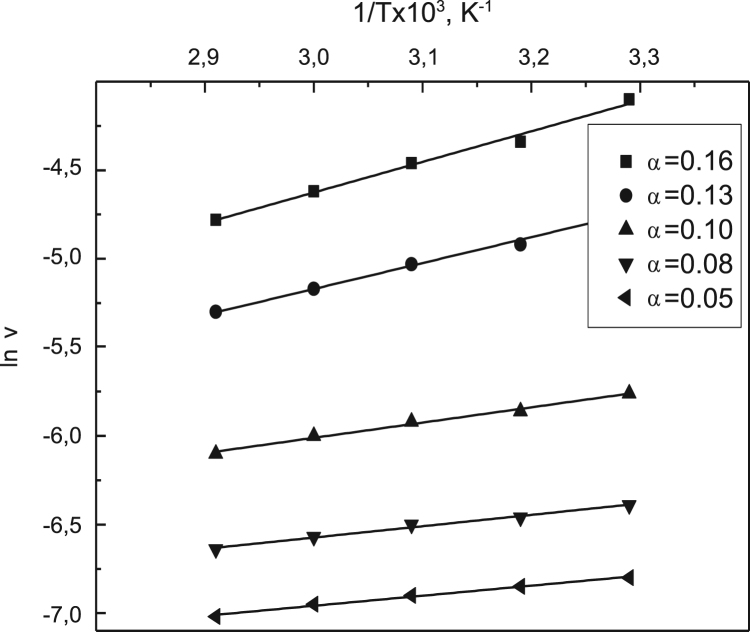
Temperature dependence of the current rate and the rate constant at α=*const* for the immobilized β-amylase.

The activity β-amylase was determined through the detection of released reducing sugars from starch using 3,5-dinitrosalicyclic acid [7].

The kinetic of the process was studied from 20 to 180 min at different increased temperatures. High temperatures were not used because the evidence of enzyme inactivation. A kinetic variable conversion degree α is used as an undimentional quantity, which could be thought as a degree of substrate conversion. Diffusion, topochemical and heterogeneous processes were examined by the meaning of the modified Prout-Tompkins topchemical equation [8], [9], [10].
