# Supplementary material for: Valorization of a plant β-amylase: Immobilization and dataset on the kinetic process
Source: Data Brief. 2017 Nov 23;16:386–91. doi: 10.1016/j.dib.2017.11.071 (PMC5723269; doi:10.1016/j.dib.2017.11.071)
Supplement: Supplementary file 1 — Supplementary material [file mmc1.doc]

**Conflict of Interest Statement**

I, Imen Lahmar, certify that I have participated sufficiently in the conception and design of this work and the analysis of the data (wherever applicable), as well as the writing of the manuscript, to take public responsibility for it. I believe the manuscript represents valid work. I have reviewed the final version of the manuscript and approve it for publication. Neither has the manuscript nor one with substantially similar content under my authorship been published nor is being considered for publication elsewhere, except as described in an attachment. Furthermore I attest that I shall produce the data upon which the manuscript is based for examination by the editors or their assignees, if requested.

I am, Imen Lahmar, the corresponding author, take full responsibility of all the above-mentioned information and I hereby accept the terms of the above Author Agreement. I am signing on behalf of all co-authors of the manuscript: “Valorization of a plant β-amylase: immobilization and dataset on the kinetic process”.
